# A Prospective, Open-Label Study to Evaluate Dual-Modality Treatment With Deoxycholic Acid (ATX-101) and Hyaluronic Acid (VYC-20L) for Overall Improvement in Jawline Contour

**DOI:** 10.1093/asjof/ojaf081

**Published:** 2025-06-28

**Authors:** Greg J Goodman, Stefania Roberts, Natasha Cook, Mark Ashton, Lucille Alker, Michael Silberberg

## Abstract

**Background:**

A poorly defined jawline and blunting of the submental angle by excess submental fat (SMF) contribute to the loss of jawline contour. The injectable adipocytolytic agent ATX-101 reduces SMF, and the hyaluronic acid filler VYC-20L restores facial volume in the chin and jaw.

**Objectives:**

The objective of this study was to evaluate the benefits of ATX-101 followed by VYC-20L for improving overall jawline contour and definition.

**Methods:**

In this Phase 4, prospective, open-label, multicenter trial, participants received 1 to 6 ATX-101 treatments (8 weeks apart), followed by VYC-20L with optional VYC-20L touch-up after 14 days. The primary endpoint was Allergan Loss of Jawline Definition Scale (ALJDS) responder rate (proportion achieving ≥1-point improvement from baseline in investigator-assessed ALJDS score 4 weeks after final VYC-20L treatment). Secondary and exploratory endpoints included clinician- and participant-assessed measures of jawline definition, satisfaction, skin laxity, and SMF. Treatment-emergent adverse events (TEAEs) were monitored.

**Results:**

Overall, 53 adults were enrolled and treated. Among 42 evaluable participants, the ALJDS responder rate was 92.9% (95% CI, 80.5-98.5). Consistent improvement across a range of clinician- and participant-assessed scales was achieved, including improvements in SMF Rating Scale score, Submental Skin Laxity Grade, and Global Aesthetic Improvement Scale score. Improvements in FACE-Q Satisfaction with Lower Face and Jawline and Appraisal of Area Under Chin indicated high levels of patient satisfaction. All participants experienced at least 1 TEAE; the majority of TEAEs were moderate in severity.

**Conclusions:**

Sequential treatment with ATX-101 and VYC-20L may be effective for improving overall jawline contour and definition, with an acceptable safety profile.

**Level of Evidence: 2 (Therapeutic):**

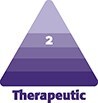

The jawline and adjoining submental area impact the overall aesthetic appearance of the face.^1-3^ Excess submental fat (SMF) detracts from jawline definition and contributes to an aged or overweight appearance, which can negatively impact self-perception.^1,4^ An American Society for Dermatologic Surgery consumer survey showed that 73% of consumers are bothered by excess fat under the chin or neck and, in another survey, 77% of participants reported noticing extra fat in the submental area and 61% wanted it reduced.^5,6^ The negative psychological impacts of excess SMF and its effect on jawline aesthetics may drive individuals to seek cosmetic correction.^1^ These data highlight the importance of jawline aesthetics and the large demand for effective methods to improve jawline definition.

Although surgical liposuction of the neck and chin (alone or as part of a platysmaplasty) can effectively address SMF and enhance existing jawline definition, it may not be a practical or preferable option for all patients.^7,8^ The availability of nonsurgical treatments for SMF reduction allows patients further options to improve their neck in conjunction with their lower face and also allows practitioners options to include the neck in conjunction with their facial analysis. Nonsurgical options for SMF reduction may serve a broader range of patients and can be employed in a multimodal treatment approach to create customizable treatment plans.^8^

Improvements in chin appearance and jawline contour can be achieved with injectable products. ATX-101 (Kybella/Belkyra; Allergan Aesthetics, an AbbVie Company, Irvine, CA) is a formulation of deoxycholic acid that causes adipocytolysis and is indicated for improvement in the appearance of moderate-to-severe convexity or fullness associated with SMF.^9-13^ After SMF reduction, dermal fillers may help further define the jawline. The choice of dermal filler is influenced by patient anatomy, product characteristics, and preference of the patient and provider.^14^ VYC-20L (Juvéderm Voluma with Lidocaine; Allergan Aesthetics) is a hyaluronic acid (HA) filler employed to restore facial volume and is approved for jawline contouring in Australia.^15,16^ Previous studies have evaluated the effectiveness of VYC-20L for structural support of the chin and for improvements in the angle of the jaw and jawline.^17,18^ Compared with properties of other HA fillers from the same manufacturer, the stiffer gel characteristics and enhanced cross-linking of VYC-20L are desirable properties for providing structure along the jaw.^16,19^

The objective of this study was to evaluate the benefits of sequential treatment with ATX-101 followed by VYC-20L for improving overall jawline contour and definition.

## METHODS

### Participants

Participants were recruited from a combination of advertisements and study investigators’ private practices. The inclusion and exclusion criteria are given in Supplemental Table 1. Briefly, enrolled participants were men and women aged 18 to 65 years with Grade ≥2 on the Allergan Loss of Jawline Definition Scale (ALJDS) on both sides of the face as determined by the investigator and Grade 2 or 3 on the Clinician-Reported Submental Fat Rating Scale (CR-SMFRS) who agreed to abstain from treatment/behavior that would affect the assessments of the submental area during the study. Participants with anatomic features that would affect assessments of the submental area (eg, BMI > 35 kg/m^2^, lower face asymmetry) were excluded, as were those with a history of filler or toxin injections or any intervention to treat SMF. All participants provided written consent for their data to be analyzed.

### Study Design

This was a Phase 4, prospective, open-label, multicenter, interventional study conducted at 3 centers in Australia from February 2018 to December 2019 (NCT03425253). The study design called for sequential treatment with ATX-101 followed by VYC-20L in order to observe the independent contributions of these agents to the final outcome.

**Figure 1.**
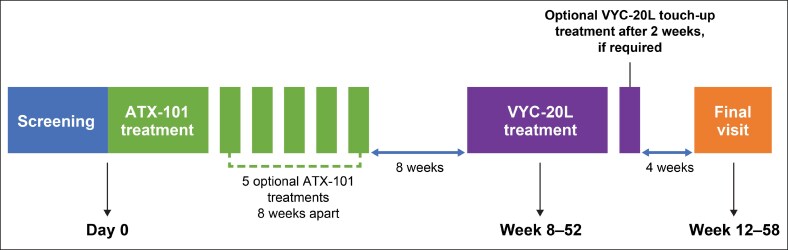
Study design.

All study treatments were performed by study investigators who were dermatologists, cosmetic physicians, or plastic surgeons (authors G.J.G., S.R., N.C., and M.A.). Participants received at least 1 ATX-101 treatment followed by up to 5 optional ATX-101 treatments at least 8 weeks apart (Figure 1). The minimum 8-week interval was chosen because the results from previous studies suggested that a 4-week interval was too short to see the final result before retreatment.^20^ For each treatment, a maximum of 50 ATX-101 injections (0.2 mL each) were spaced 1 cm apart in the subcutaneous fat tissue of the submental area to attain a dose of 2 mg/cm^2^. Maximum total volumes of 10 mL per treatment visit and 60 mL over 6 visits were permitted. An injection grid was employed to outline the planned treatment area and to mark the injection sites. The injection sites were marked by outlining the planned treatment area with a surgical pen and a 1 cm injection grid, avoiding the area of the marginal mandibular nerve. The participant was asked to tense the platysma. While pinching the SMF, 0.2 mL of ATX-101 was injected into the preplatysmal fat next to each marked injection site by a 30 G or smaller, 0.5-inch needle, advancing the needle perpendicular to the skin. Superficial (ie, into the dermis) injections were avoided to prevent skin ulceration. ATX-101 was injected at a depth of approximately midway into the subcutaneous fat layer to avoid injection into the postplatysmal fat; muscle, salivary glands, and lymph nodes were also avoided. The needle was not withdrawn from the subcutaneous fat during injection to prevent intradermal exposure and skin ulceration. However, the needle was withdrawn to an appropriate depth before injection if resistance (indicating possible contact with fascial or nonfat tissue) was met. Upon needle withdrawal, pressure was applied to the injection site to minimize bleeding, and an adhesive dressing was applied as needed. Oral analgesics or nonsteroidal anti-inflammatory drugs, topical and/or injectable local anesthesia (eg, lidocaine), and/or cooling packs were allowed at the area of injection (for treatment or biopsy) at the discretion of the investigator/injector. The injected area was massaged to ensure uniform distribution of ATX-101.

Eight weeks after each ATX-101 treatment, the investigator and participant evaluated whether desired results had been achieved and discussed whether additional ATX-101 treatments were required, at the final discretion of the investigator. After it was determined that further ATX-101 intervention was not required or the maximum of 6 ATX-101 treatments had been administered, participants received VYC-20L along the mandibular border with an optional touch-up treatment after 14 days. The maximum total volume allowed for the initial and touch-up treatments combined was 6 mL (3 mL per side of the face). Depending on the investigator's judgment, VYC-20L was administered into the supraperiosteal plane by a needle or cannula per Allergan guidance. After insertion of the needle or cannula but before injection, the plunger was withdrawn to aspirate and verify that the needle or cannula was not intravascular. The injected volume of VYC-20L was distributed between the right and left sides of the face according to the needs of each area. After the injection, the treated area was massaged to ensure uniform distribution of VYC-20L.

The study design was conducted in compliance with the guidelines on Good Clinical Practice and approved by a central ethics committee (Bellberry Human Research Ethics Committee, Eastwood, South Australia, Australia).

### Effectiveness Endpoints

Details of the endpoints assessed in this study and scale descriptions are given in Supplemental Table 2. The primary endpoint was ALJDS responder rate, defined as the proportion of participants achieving ≥1-point improvement from baseline in the ALJDS score (average of both sides of the face) at the final study visit (4 weeks after final VYC-20L treatment to allow sufficient time to observe final results) as assessed by the investigator. Investigator assessments of ALJDS were performed by study investigators and other trained clinical staff.

Secondary endpoints included ALJDS responder rate and change from baseline in the ALJDS score as assessed by an independent reviewer, changes in the validated CR-SMFRS and Patient-Reported Submental Fat Rating Scale (PR-SMFRS), Submental Skin Laxity Grade (SMSLG) score, and validated FACE-Q scale scores for jawline, lower face, and chin assessment.^10,21-23^ Independent reviewer assessments were based on standardized photographs captured by trained study staff using a VECTRA M3 camera system (Canfield Scientific). Exploratory endpoints assessed change from baseline to 8 weeks after last ATX-101 treatment on the CR-SMFRS, PR-SMFRS, SMSLG, and FACE-Q scores as well as Global Aesthetic Improvement Scale (GAIS) scores (Supplemental Table 2). Total follow-up time could range from 12 to 58 weeks, depending on the number of ATX-101 treatments received.

### Safety

Adverse events (AEs) were monitored throughout the study. Participants recorded injection site reactions in a 28-day diary during the VYC-20L treatment period.

### Statistical Analysis and Analysis Populations

Statistical analyses were performed by SAS version 9.4 or higher (SAS Institute Inc, Cary, NC). Effectiveness endpoints were summarized descriptively with 95% CIs, and statistical differences from baseline in ALJDS, CR-SMFRS, PR-SMFRS, and SMSLG scores were analyzed by paired *t* test (or Wilcoxon’s signed-rank test if normality assumptions were not met). FACE-Q scores were presented as Rasch-transformed scores (0 [worst] to 100 [best]), and differences from baseline were analyzed by paired *t* tests. Data were pooled across clinical centers for all analyses.

Three analysis populations were employed for statistical analysis. The primary endpoint was analyzed in the evaluable set, which comprised participants who received both study treatments (ATX-101 and VYC-20L) and had 1 posttreatment effectiveness assessment. Other effectiveness endpoints were analyzed in the full analysis set, which comprised participants who received at least 1 ATX-101 treatment and 1 posttreatment effectiveness assessment. Safety outcomes were evaluated in the safety population, which included participants who consented to participate in the study and met eligibility criteria.

## RESULTS

### Participants

Of 58 enrolled participants, 53 met eligibility criteria (safety population). Most participants were female (*n*/*N* = 44/53 [83.0%] vs *n*/*N* = 9/53, [17.0%] males) and White (96.2% [*n* = 51]), with a mean age of 48.0 years (range, 24-64 years) and a mean (standard deviation [SD]) BMI of 28.0 (3.53) kg/m^2^ (Table 1). Of the 53 eligible participants, all received at least 1 ATX-101 treatment and 1 posttreatment effectiveness assessment (full analysis set), and 42 (79.2%) received both study treatments and 1 posttreatment effectiveness assessment (evaluable set); all participants in the evaluable set completed the study. Eleven participants discontinued during the study owing to withdrawal of consent (*n* = 6), loss to follow-up (*n* = 3), and withdrawal by investigator owing to AEs (*n* = 2). Of the 6 participants who withdrew consent, 1 did so owing to an AE.

**Table 1.** Participant Demographics and Baseline Data

Parameter	*N* = 53
Sex, *n* (%)	
Female	44 (83.0)
Male	9 (17.0)
Age, mean (SD), years	48.0 (11.1)
Age range, years	24-64
Race, *n* (%)	
White; not Hispanic or Latino	51 (96.2)
Asian	1 (1.9)
Other	1 (1.9)
Weight, mean (SD), kg	79.4 (13.5)
Weight range, kg	51.0-108.0
BMI, mean (SD), kg/m^2^	28.0 (3.5)
BMI range, kg/m^2^	20.0-34.6
Fitzpatrick skin type, *n* (%)	
I-III	51 (96.2)
IV-VI	2 (3.8)
Baseline ALJDS, left side, right side, *n* (%)	
Grade 2 (moderate)	20 (37.7), 24 (45.3)
Grade 3 (severe)	32 (60.4), 29 (54.7)
Grade 4 (extreme)	1 (1.9), 0 (0)
Baseline CR-SMFRS, *n* (%)	
Grade 3 (severe)	32 (60.4)
Grade 4 (extreme)	21 (39.6)
Baseline SMSLG, *n* (%)	
Grade 1 (none)	13 (24.5)
Grade 2 (mild)	32 (60.4)
Grade 3 (moderate)	8 (15.1)

ALJDS, Allergan Loss of Jawline Definition Scale; CR-SMFRS, Clinician-Reported Submental Fat Rating Scale; SD, standard deviation; SMSLG, Submental Skin Laxity Grade.

### Treatment Sessions

During the study, a total mean (SD) of 5.2 (2.3) mL of ATX-101 was injected per treatment session. On average (SD), participants received 4.9 (1.5) sessions with ATX-101 and duration of treatment was 237.3 (94.1; range, 1-404) days. A total mean (SD) of 2.4 (1.2) mL of VYC-20L was injected (distributed between both sides of the face) for both initial and touch-up treatments combined. On average (SD), participants received 1.9 (0.3) treatment sessions with VYC-20L and duration of treatment was 18.4 (9.4; range, 1-6) days. The mean follow-up time for participants who completed the study was 388.5 (SD, 90.0; range, 163-511) days.

### Primary Endpoint

**Figure 2.**
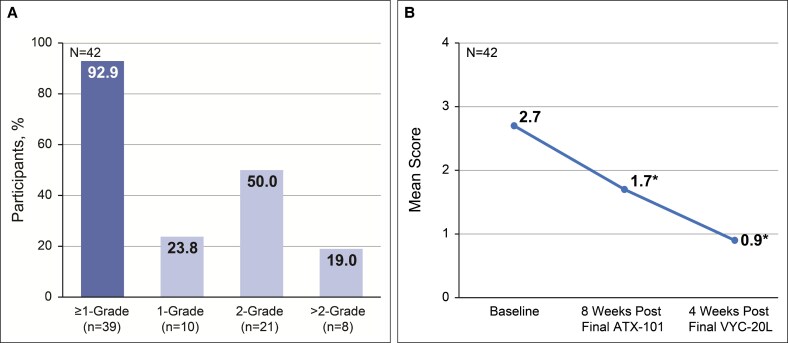
Investigator-assessed Allergan Loss of Jawline Definition Scale (ALJDS). (A) Proportion of participants achieving improvement on the ALJDS. (B) Mean ALJDS score from baseline to final study visit (4 weeks after final VYC-20L treatment). Lower scores indicate improvement in jawline definition. *P < .001 vs baseline. ALJDS is based on the average of both the left and right sides of the face. ALJDS grades: 0 (none), 1 (mild), 2 (moderate), 3 (severe), and 4 (extreme).

Of 42 participants, 39 (92.9%; 95% CI, 80.5-98.5) demonstrated a ≥1-point improvement from baseline to final study visit in the investigator-assessed ALJDS (Figure 2A).

### Secondary Endpoints

**Figure 3.**
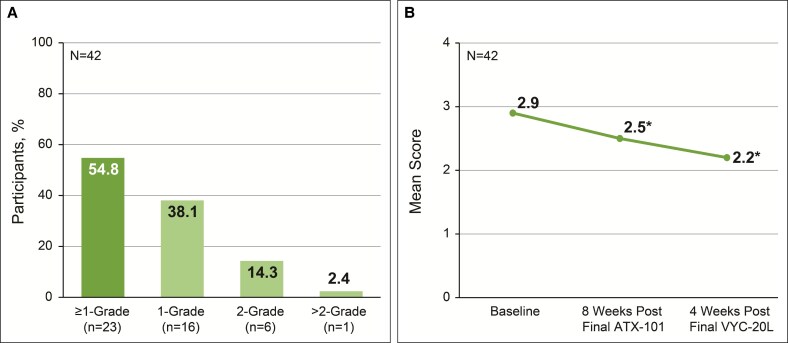
Independent reviewer–assessed Allergan Loss of Jawline Definition Scale (ALJDS). (A) Proportion of participants achieving improvement on the ALJDS. (B) Mean ALJDS score from baseline to final study visit (4 weeks after final VYC-20L treatment). Lower scores indicate improvement in jawline definition. *P < .005 vs baseline. ALJDS is based on the average of both the left and right sides of the face. ALJDS grades: 0 (none), 1 (mild), 2 (moderate), 3 (severe), and 4 (extreme).

The mean improvement from baseline on the ALJDS was −1.0 point (95% CI, −1.2 to −0.7; *P* < .001) at 8 weeks after final ATX-101 treatment and −1.8 points (95% CI, −2.1 to −1.5; *P* < .001) at 4 weeks after final VYC-20L treatment (Figure 2B). The proportion of participants achieving a ≥1-point improvement from baseline to final study visit in the independent reviewer–assessed ALJDS included 23 of 42 participants (54.8%; 95% CI, 38.7-70.2; Figure 3A). The mean improvement from baseline was −0.4 points (95% CI, −0.7 to −0.1; *P* = .004) at 8 weeks after final ATX-101 treatment and −0.7 points (95% CI, −1.0 to −0.4; *P* < .001) at 4 weeks after final VYC-20L treatment (Figure 3B).

**Figure 4.**
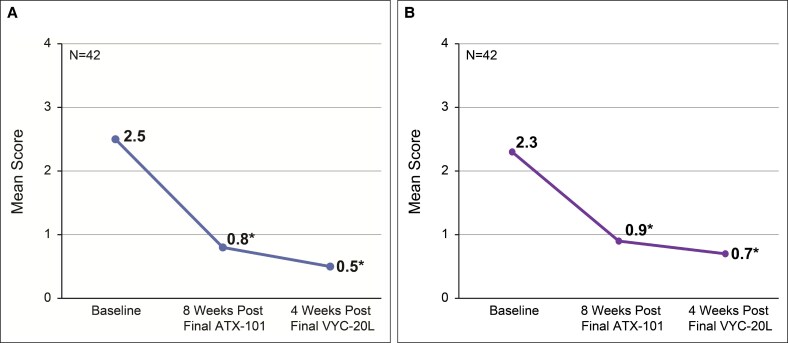
Mean (A) Clinician-Reported Submental Fat Rating Scale (CR-SMFRS) and (B) Patient-Reported Submental Fat Rating Scale (PR-SMFRS) scores from baseline to final study visit (4 weeks after final VYC-20L treatment). Lower scores indicate improvement. *P < .001 vs baseline. CR-SMFRS grades: 0 (absent), 1 (mild), 2 (moderate), 3 (severe), and 4 (extreme). PR-SMFRS grades: 0 (no chin fat at all), 1 (slight amount of chin fat), 2 (moderate amount of chin fat), 3 (large amount of chin fat), and 4 (a very large amount of chin fat).

**Figure 5.**
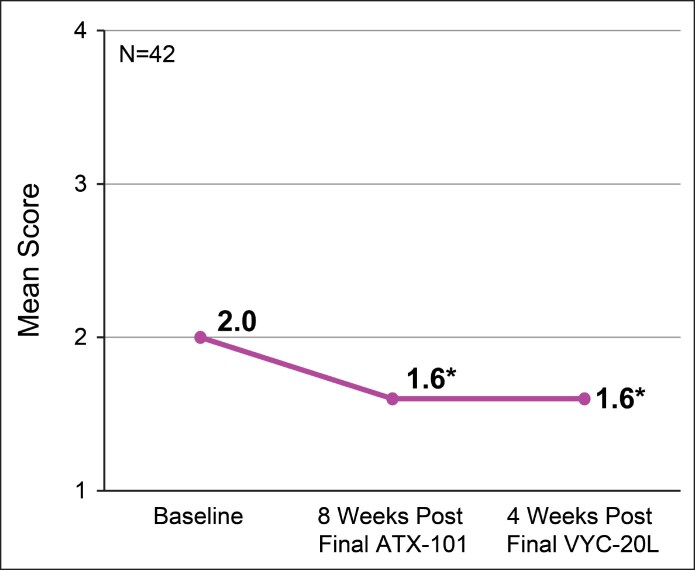
Mean Submental Skin Laxity Grade (SMSLG) scores from baseline to final study visit (4 weeks after final VYC-20L treatment). Lower scores indicate improvement. *P ≤ .005 vs baseline. SMSLG scale: 1 (none), 2 (mild), 3 (moderate), and 4 (severe).

At 4 weeks after final VYC-20L treatment, the mean improvement from baseline in the CR-SMFRS was −1.9 (95% CI, −2.2 to −1.7; *P* < .001; Figure 4A). The mean improvement in PR-SMFRS was −1.6 (95% CI, −1.9 to −1.3; *P* < .001; Figure 4B), and in SMSLG was −0.3 points (95% CI, −0.5 to −0.1; *P* = .003) from baseline to final study visit at 4 weeks after final VYC-20L treatment (Figure 5).

**Figure 6.**
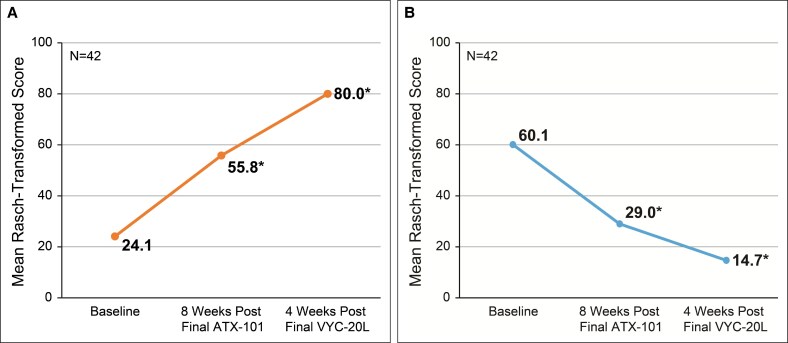
Mean Rasch-transformed FACE-Q. (A) Satisfaction with Lower Face and Jawline and (B) Appraisal of Area Under Chin scores from baseline to final study visit (4 weeks after final VYC-20L treatment). Higher scores for the Satisfaction with Lower Face and Jawline and lower scores for the Appraisal of Area Under Chin indicate improvement. *P < .001 vs baseline. Satisfaction with Lower Face and Jawline scores range from 1 (very dissatisfied) to 4 (very satisfied). Appraisal of area under chin scores range from 1 (not at all) to 4 (extremely). Rasch-transformed scores range from 0 (worst) to 100 (best).

The mean improvement from baseline in FACE-Q score for the Satisfaction with Lower Face and Jawline scale was +55.9 points (95% CI, 48.6-63.3; *P* < .001) at 4 weeks after final VYC-20L treatment (Figure 6A). The mean improvement from baseline in the FACE-Q score for the Appraisal of Area Under Chin scale was −45.4 points (95% CI, −54.5 to −36.3; *P* < .001) at 4 weeks after final VYC-20L treatment (Figure 6B).

### Exploratory Endpoints

The mean improvement from baseline in the CR-SMFRS was −1.7 points (95% CI, −1.8 to −1.5; *P* < .001) at 8 weeks after final ATX-101 treatment (Figure 4A). The mean improvement from baseline in PR-SMFRS was −1.4 points (95% CI, −1.7 to −1.2; *P* < .001) at 8 weeks after final ATX-101 treatment (Figure 4B). The mean improvement from baseline in SMSLG was −0.3 points (95% CI, −0.6 to −0.1; *P* = .005) at 8 weeks after final ATX-101 treatment (Figure 5).

At 8 weeks after final ATX-101 treatment, the mean improvement from baseline in FACE-Q score for the Satisfaction with Lower Face and Jawline scale was +31.7 points (95% CI, 23.0-40.3; *P* < .001; Figure 6A), and the mean improvement from baseline in the FACE-Q score for the Appraisal of Area Under Chin scale was −31.1 points (95% CI, −42.3 to −19.8; *P* < .001; Figure 6B).

**Figure 7.**
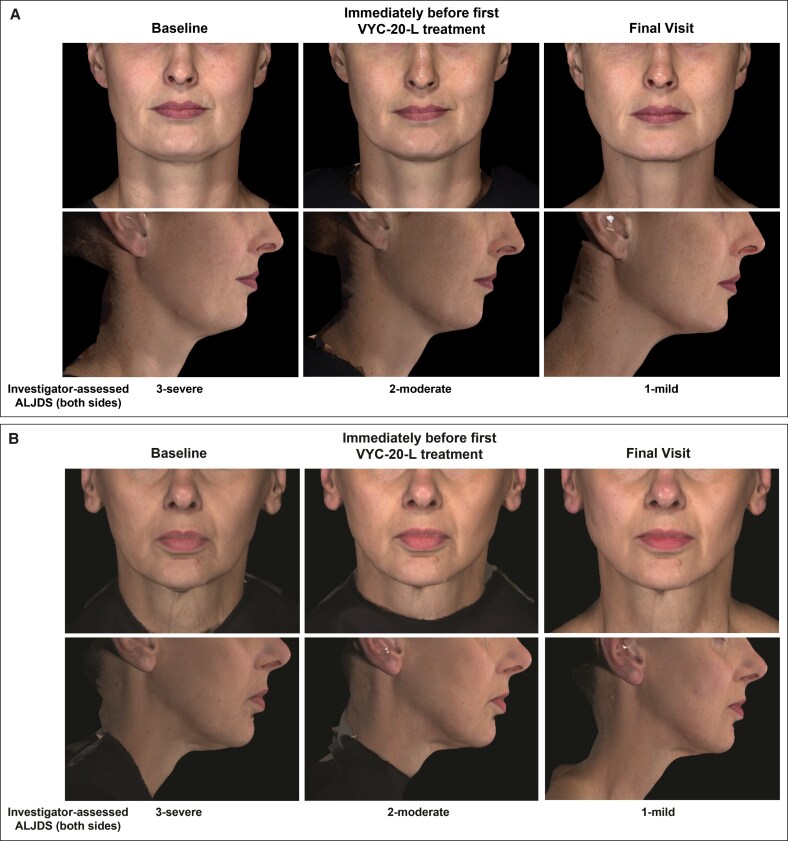
Representative photographs of participants showing a ≥2-point improvement on the Allergan Loss of Jawline Definition Scale (ALJDS). (A) A 45-year-old female participant (BMI, 24.8 kg/m^2^) was injected with a mean of 8.2 (SD, 0.35; range, 8.0-8.6) mL of ATX-101 per treatment session (1 initial and 2 additional treatment sessions), followed by a total of 4.0 and 2.0 mL of VYC-20L for both sides of the face for initial and touch-up treatments, respectively (combined total of 6.0 mL). (B) A 57-year-old female participant (BMI, 21.4 kg/m^2^) was injected with a mean of 7.0 (SD, 1.73; range, 5.0-8.0) mL of ATX-101 per treatment session (1 initial and 2 additional treatment sessions), followed by a total of 5.0 and 1.0 mL of VYC-20L for both sides of the face for initial and touch-up treatments, respectively (combined total of 6.0 mL). Injected volumes of VYC-20L were distributed between the right and left sides of the face according to the needs of each area.

The mean investigator- and participant-assessed GAIS scores were 1.6 and 1.7 (2 = much improved), respectively, at 8 weeks after final ATX-101 treatment. The mean investigator- and participant-assessed GAIS scores were 1.7 and 1.8, respectively, at 4 weeks after final VYC-20L treatment. Representative photographs illustrating changes from baseline in the lower facial appearance of 2 study participants are shown in Figure 7.

### Safety

All participants (*N* = 53) reported at least 1 treatment-emergent AE (TEAE), and the majority of TEAEs were mild to moderate in severity (69.8%). All 53 participants (100%) reported at least 1 TEAE related to ATX-101, and 3 of 53 participants (5.7%) reported at least 1 TEAE related to VYC-20L (Table 2). Two (3.8%) of 53 participants experienced a total of 5 serious AEs (SAEs), which were unrelated to treatments or study procedures. One participant experienced an SAE of lower respiratory tract infection, and 1 participant experienced SAEs of severe volvulus, uterine leiomyoma, and vaginal hemorrhage (2 dates). No participants were discontinued from treatment owing to an SAE.

**Table 2.** Treatment-Emergent Adverse Events (TEAEs) Related to ATX-101 and VYC-20L Treatment (*N* = 53)

TEAEs	Related to ATX-101, *n* (%)^a^	Related to VYC-20L, *n* (%)
IS pain	39 (73.6)	1 (1.9)
IS swelling	39 (73.6)	0
IS bruising	36 (67.9)	2 (3.8)
IS mass	30 (56.6)	0
IS hypoesthesia	29 (54.7)	0
IS induration	24 (45.3)	0
IS reaction	11 (20.8)	0
IS erythema	11 (20.8)	0
Contusion	8 (15.1)	0
IS discomfort	8 (15.1)	1 (1.9)
IS warmth	8 (15.1)	0
IS discoloration	7 (13.2)	0
Headache	7 (13.2)	0
Swelling face	6 (11.3)	0
IS pruritus	5 (9.4)	0
Swelling	4 (7.5)	0
Weight decreased	3 (5.7)	0
Nausea	3 (5.7)	0
Alopecia	2 (3.8)	0
Postprocedural contusion	2 (3.8)	0
IS paresthesia	2 (3.8)	0

IS, injection site; TEAE, treatment-emergent adverse event.

^a^TEAEs related to ATX-101 in >1 participant.

Of the 53 participants who experienced TEAEs related to ATX-101, the most common were injection site pain (39, 73.6%), injection site swelling (39, 73.6%), and injection site bruising (36, 67.9%; Table 2). Ten (18.9%) of 53 participants reported the following complications related to ATX-101: contusion (8, 15.1%), postprocedural contusion (2, 3.8%), and procedural pain (1, 1.9%). Three (5.7%) of 53 participants discontinued the study owing to ATX-101-related AEs. Of these 3 participants, 1 elected to withdraw consent from the study owing to procedural pain and the other 2 were withdrawn by the investigator because of injection site–related AEs, including discoloration, induration, pain, and rash. The most common TEAE related to VYC-20L was injection site bruising (2/53, 3.8%; Table 2). No participants reported a complication related to VYC-20L.

## DISCUSSION

The jawline plays a prominent role in the facial profile.^1-3^ During aging, the jawline loses definition as a result of structural, bony, soft tissue, and skin changes.^24^ These include an increasing cervicomental angle (an angle between the vertical portion of the neck and the transverse portion of the submandibular region), mandible recession, SMF accumulation, weakening of the mandible septum (which holds fat compartments in place), and skin laxity (which leads to sagging jowl fat).^1,24^ The change in the appearance of the jawline over time may cause feelings of embarrassment and low self-esteem.^1^ The negative psychological impact of excess SMF underlines the importance of optimizing treatment options for jawline rejuvenation.

Here, we report the results of a nonsurgical, multimodal approach to improve jawline aesthetics. This study demonstrates that sequential, noninvasive treatment by ATX-101 followed by VYC-20L improves the jawline contour by reducing submental fullness and enhancing mandibular border definition, respectively. The primary endpoint was met, with 92.9% of participants achieving a ≥1-point improvement in jawline definition from baseline to final study visit using the ALJDS scale. Consistent improvement across a range of clinician- and participant-assessed scales was achieved, including a high level of satisfaction with treatment results. In addition, skin laxity was improved following reduction in SMF, which supports the findings of previous studies with ATX-101.^12,21^ These data support the effectiveness of these 2 agents in improving jawline contour to help patients achieve a satisfactory facial aesthetic.

Several studies investigating the effectiveness of ATX-101 and HA fillers individually on the chin and jawline have been conducted. ATX-101 treatment has been shown to be safe and effective in reducing SMF and resulted in increased patient satisfaction with facial appearance.^9-12^ Similarly, HA filler injections have demonstrated effectiveness in chin and jawline enhancement with high levels of patient satisfaction.^18,25,26^ Combination treatment has also shown promise in early studies: sequential treatment by ATX-101 and HA fillers improved overall facial appearance and perceived age in addition to having psychosocial benefits.^27^ Panfacial aesthetic treatment by different minimally invasive modalities is recognized as a valid approach to optimizing treatment outcomes.^28^

Treatment with ATX-101 and VYC-20L was well tolerated in the present study. AEs and injection site reactions, including swelling, which is an expected part of the recovery period, were consistent with studies using ATX-101 or HA fillers individually for chin and jawline reshaping.^9-13,18,25,26^ The majority of TEAEs were mild to moderate in severity and involved the injection site.

The study design has limitations. Investigator-conducted assessments are subjective and may be influenced by individual biases. Responder rates based on investigator-assessed ALJDS scores were substantially higher than those based on independent reviewer–assessed scores (92.9% vs 54.8%, respectively) and were in line with large improvements from baseline on participant-assessed scales, indicating that live investigator assessments may have resulted in more positive ratings vs the static photograph assessments conducted by the independent reviewer and/or that the open-label study design may have led to more positive responses from the evaluating investigators and participants compared with a blinded study design. Previous studies of ATX-101 for SMF reduction analyzed the effects of treatment by magnetic resonance imaging.^9,10^ The current study did not include supporting radiological assessments to measure effectiveness. The number of treatments with ATX-101 and VYC-20L was higher than the number of anticipated treatments based on clinical experience, which may have been owing to the cost of treatment being covered by the study sponsor. In real-world practice, it is possible that treatment costs would contribute to limiting the number of treatments sought. Furthermore, the current study only evaluated dual-modality treatment with ATX-101 and VYC-20L and did not assess other means of surgical (eg, liposuction) and nonsurgical SMF reduction (eg, cryolipolysis), or other HA fillers, such as VYC-25L (Juvéderm Volux with Lidocaine; Allergan Aesthetics), which has been approved for restoring and creating volume in the chin and jawline area.^26^ Comparisons of ATX-101 and VYC-20L with other dual-modality approaches may be considered for future studies. The study population was mostly White and female, limiting generalizability to other racial groups and males. In addition to increasing gender and racial diversity, future studies may consider subanalyses by age, gender, and skin type. The discontinuation rate among the 53 participants who received treatment (11, 19%) may also limit the ability to extrapolate the study findings to the general population. Although 3 participants discontinued owing to AEs (1 of whom was categorized as having withdrawn consent because of procedural pain), none discontinued owing to an SAE. A larger, longer-term study may be needed to provide a full assessment of the duration of effectiveness and safety.

Overall, this study demonstrated the benefit of combined treatment with ATX-101 followed by VYC-20L for improving jawline aesthetics, enhancing a growing trend in the aesthetic field of using multimodal treatment approaches that consider both the face/jawline and submental area.^28^ This study investigates minimally invasive treatment options for patients, especially those who are not suitable candidates for, or are unwilling to undergo, traditional invasive procedures for SMF reduction, which have the potential for postprocedural complications.

## CONCLUSIONS

Sequential treatments of ATX-101 and VYC-20L enhanced the overall contour of the jawline by addressing convexity or fullness associated with SMF and restoring volume along the mandible, respectively, with an acceptable safety profile. Participants were satisfied with the results of this multimodal treatment approach on validated outcome measures.

The clinical trial data associated with this article can be requested online by qualified researchers through Vivli (Burlington, MA) following review and approval of a research proposal, statistical analysis plan, and execution of a data sharing agreement. The data will be accessible for 12 months.

## Supplemental Material

This article contains supplemental material located online at https://doi.org/10.1093/asjof/ojaf081.

## Supplementary Material

ojaf081_Supplementary_Data
