# Supplementary material for: A Prospective, Open-Label Study to Evaluate Dual-Modality Treatment With Deoxycholic Acid (ATX-101) and Hyaluronic Acid (VYC-20L) for Overall Improvement in Jawline Contour
Source: Aesthet Surg J Open Forum. 2025 Jun 28;7:ojaf081. doi: 10.1093/asjof/ojaf081 (PMC12342753; doi:10.1093/asjof/ojaf081)
Supplement: ojaf081_Supplementary_Data [file ojaf081_supplementary_data.docx]

# **Supplemental Material**

**Supplemental Table 1. Key Inclusion and Exclusion Criteria**

| **Inclusion Criteria** |
| --- |
| - Males and females 18 to 65 years of age - Grade ≥2 on the Allergan Loss of Jawline Definition Scale (0=none, 4=extreme), on both sides of the face as determined by the investigator - Grade 2 or 3 on the Clinician-Reported Submental Fat Rating Scale (0=absent, 4=extreme) - Stable body weight for ≥26 weeks prior to study - Agreement to abstain from treatment/behavior that would affect the assessments of the submental area during the study (eg, unshaven facial hair, changes to dietary/exercise habits) |
| **Exclusion Criteria** |
| - Grade 4 on the 4-point Submental Skin Laxity Grade scale (1=none, 4=severe) or other anatomical feature that would affect assessments of the submental area (eg, predominant subplatysmal fat, loose skin in the neck/chin) - Grade 4 on the 5-point Allergan Jowl Fat Rating Scale (0=absent/minimal, 1=mild, 2=moderate, 3=severe, 4=extreme) - Body mass index >35 kg/m^2^ - Asymmetry in the lower face per investigator judgment - Any previous intervention to reduce submental fat - History of trauma or presence of lesions associated with the submental area/neck - Clinically significant bleeding disorder - History of or plans to receive facial/neck filler or botulinum toxin injections in the lower face; ablative procedures, skin resurfacing, plastic surgery, tissue grafting, or implants in the face/neck area during the study; systemic retinoid therapy; anticoagulation therapy; oral corticosteroid therapy; or oral surgery/dental procedures within 2 weeks prior to and after VYC-20L treatment - Pregnant, lactating, or planning to become pregnant during the study |

**Supplemental Table 2. Scales Used for Effectiveness Endpoints**

| **Scale** | **Evaluator** | **Endpoint** | **Assessment** | **Measure** |
| --- | --- | --- | --- | --- |
| Allergan Loss of Jawline Definition Scale (ALJDS) | Investigator | *Primary*: The proportion of subjects who show ≥1 point improvement from baseline at the final study visit (4 weeks after last VYC-20L treatment) assessed live (ie, responder rate) | The ALJDS assesses jawline contour via a 5-point scale on both sides of the face. | 0 = None  1 = Mild  2 = Moderate  3 = Severe  4 = Extreme |
| ALJDS | Investigator | *Secondary*: Mean change from baseline to last ATX-101 treatment | See above | See above |
| ALJDS | Independent reviewer | *Secondary*: The proportion of subjects who show ≥1 point improvement from baseline at the final study visit (4 weeks after last VYC-20L treatment) assessed via photographic images | See above | See above |
| ALJDS | Independent reviewer | *Secondary*: Mean change from baseline to last ATX-101 treatment and final study visit via photographic images | See above | See above |
| FACE-Q Satisfaction with Lower Face and Jawline | Participant | *Secondary*: Mean change from baseline to final study visit in self-appraisal of jawline and chin satisfaction | In the past week, how satisfied or dissatisfied have you been with:   1. How prominent your jawline looks? 2. How sculpted (well defined) your jawline looks? 3. How your jawline looks in profile (side view?) 4. How nice your lower face looks? 5. How smooth your lower face looks (ie, no jowls or folds of fatty skin)? | 1 = Very dissatisfied  2 = Somewhat dissatisfied  3 = Somewhat satisfied  4 = Very satisfied |
| FACE-Q Satisfaction with Lower Face and Jawline | Participant | *Exploratory*: Change from baseline to 8 weeks after last ATX-101 treatment | See above | See above |
| FACE-Q Appraisal of Area Under Chin | Participant | *Secondary*: Mean change from baseline to final study visit in self-appraisal of area under the chin | In the past week, how much have you been bothered by:   1. Fullness under your chin (eg, double chin)? 2. Lack of contour (outline under your chin)? 3. Sagging of the skin and fat under your chin? 4. Loose skin and fat under your chin? 5. How the area under your chin looks in profile (side view)? | 1 = Not at all  2 = A little  3 = Moderately  4 = Extremely |
| FACE-Q Appraisal of Area Under Chin | Participant | *Exploratory*: Change from baseline to 8 weeks after last ATX-101 treatment | See above | See above |
| Clinician-Reported Submental Fat Rating Scale (CR-SMFRS) | Investigator | *Secondary*: Mean change from baseline to final study visit assessed live | The CR-SMFRS score is based on the investigator’s clinical evaluation of the patient, including palpation of the chin and neck area; anterior, oblique, and profile views of the chin and neck; as well as observation of pronation, supination, and lateral movement of the head. The score is determined using the definitions in the rating scale and representative photographs associated with each score. | 0 = Absent submental convexity: no localized submental fat evident  1 = Mild submental convexity: minimal, localized submental fat  2 = Moderate submental convexity: prominent, localized submental fat  3 = Severe submental convexity: marked, localized submental fat  4 = Extreme submental convexity |
| CR-SMFRS | Investigator | *Exploratory*: Change from baseline to 8 weeks after last ATX-101 treatment | See above | See above |
| Patient-Reported Submental Fat Rating Scale (PR-SMFRS) | Participant | *Secondary*: Mean change from baseline to final study visit as assessed using a mirror | Looking in a mirror: how much fat do you have under your chin right now? | 0 = No chin fat at all  1 = A slight amount of chin fat  2 = A moderate amount of chin fat  3 = A large amount of chin fat  4 = A very large amount of chin fat |
| PR-SMFRS | Participant | *Exploratory*: Change from baseline to 8 weeks after last ATX-101 treatment | See above | See above |
| Submental Skin Laxity Grade (SMSLG) score | Investigator | *Secondary*: Mean change from baseline to final study visit on the SMSLG as assessed live by the investigator | SMSLG integrates 3 skin features: skin wrinkling, adherence to underlying neck structures (bone and muscle), and redundancy (horizontal and vertical folds). Each grade defines the maximal allowed limit for skin wrinkling, adherence to underlying structures, and redundancy. | 1 = None  2 = Mild  3 = Moderate  4 = Severe |
| SMSLG | Investigator | *Exploratory*: Change from baseline to 8 weeks after last ATX-101 treatment | See above | See above |
| Global Aesthetic Improvement Scale (GAIS) | Investigator | *Exploratory*: Mean score at 8 weeks after last ATX-101 treatment assessed live | Level of improvement to appearance while viewing baseline | 2 = Much improved  1 = Improved  0 = No change  −1 = Worse  −2 = Much worse |
| GAIS | Participant | *Exploratory*: Mean score at 8 weeks after last ATX-101 treatment assessed live | Level of improvement to the lower face and jawline compared with baseline image | See above |
